# Solutions for atypical problems in the incisors area: a transdisciplinary challenge

**DOI:** 10.1590/2177-6709.25.2.086-102.sar

**Published:** 2020

**Authors:** Márcio Costa Sobral

**Affiliations:** 1 Universidade Federal da Bahia, Dental School, Specialization Program in Orthodontics (Bahia, Brazil).

**Keywords:** Orthodontics, Dental aesthetics, Smile

## Abstract

**Introduction:**

A significant increase in the number of adults in search of orthodontic treatment has raised a new challenge for orthodontists: the need to interact with other specialties to achieve excellent results, particularly when dealing with smile aesthetics and facial balance. Several factors should be considered to respond to their demand: adequate tooth leveling and alignment, individual tooth proportions between adjacent teeth and their contralateral teeth, shape and natural appearance of each tooth and gingival architecture, which should all be in agreement with facial harmony. Maxillary or mandibular incisors congenitally missing or lost due to caries or trauma and tooth-size discrepancies (Bolton) are some of the important aesthetic challenges for an integrated orthodontic treatment.

**Objectives:**

This study describes cases that illustrate the clinical challenges of treating the anterior area, as well as the multidisciplinary strategies required for their resolution.

**Conclusion:**

The increasingly frequent multidisciplinary orthodontic treatments of complex cases seem to effectively maximize aesthetic and functional results using a combination of procedures conducted by specialists in related areas, such as Surgery, Prosthetics, Implantology, Restorative Dentistry and Periodontics.

## RESUMO

**Introdução:**

O aumento significativo de indivíduos adultos em busca de tratamento ortodôntico resultou em um novo desafio para o ortodontista: a necessidade de interação com outras especialidades, visando a excelência de resultados, principalmente quando o assunto é a estética do sorriso e o equilíbrio facial. Para atender a essa demanda, diversos fatores devem ser considerados: correto alinhamento e nivelamento dos dentes, proporções dentárias individuais entre dentes adjacentes e seus homólogos, forma e naturalidade de cada dente e arquitetura gengival - estando todos esses em consonância com a harmonia da face. Casos envolvendo ausência congênita ou perda de incisivos por cárie ou trauma, discrepância de tamanho dentário (Bolton), entre outras, constituem importantes desafios estéticos para o tratamento integrado em Ortodontia.

**Objetivos:**

apresentar casos que ilustram tais desafios envolvendo a região anterior, onde a abordagem transdisciplinar foi fundamental para sua resolução.

**Conclusão:**

O tratamento ortodôntico transdisciplinar, cada vez mais frequente, vem se mostrando eficiente na resolução de casos complexos, visando maximizar os resultados estéticos e funcionais, mediante a combinação de procedimentos realizados por especialidades afins, como a Cirurgia, Prótese, Implantodontia, Dentística e Periodontia.

## MISSING TEETH IN THE ANTERIOR AREA

### Agenesis, trauma

Orthodontics is a specialty that combines knowledge about biological, mechanical and artistic factors. Some conditions require a maximum interaction and use of this broad knowledge. Some examples of that are cases of missing teeth that have to be replaced in the anterior area, in the aesthetic zone, as illustrated in the cases described here. These teeth may be replaced using different techniques, such as orthodontic movement of other teeth, autotransplantation and use of fixed or implant-supported prosthesis. Several aesthetic factors, such as symmetry, morphology, shade, width, length, angulation, thickness and gingival architecture of the replaced teeth, have to be considered when planning the treatment^3^.

The ideal proportion of a tooth may be calculated as the width-to-length ratio. A maxillary central incisor (MCI) has a pleasing proportion when its width is 75%-85% of its length^4^. Another fundamental factor is the visibility of teeth in a frontal view of the smile. Claman et al.^1^ found that only 17% of the patients had a dental golden proportion, that is, when 100% of MCI are seen in a frontal view, the anteriorly visible width of maxillary lateral incisors (MLI) should be about 62% of MCI, and of canines, 62% of MLI. Therefore, the linear width of MLI should be about 2 mm shorter than that of MCI; and of canines, 1 mm shorter than that of MLI1. 

When a tooth has to be replaced, some considerations should be included in treatment planning, such as: patient age and skeletal maturation at the time agenesis was diagnosed or tooth was lost, the amount of time from orthodontic treatment to definitive restoration, and stability or longevity of results^3^.

### MLI agenesis

The relatively common MLI agenesis, one of the most frequent types of agenesis in permanent dentition, as well as other atypical morphology abnormalities, substantially compromises smile aesthetics. Its frequency varies according to the characteristics of the population under study and the sex of the participants, and values range from 0.8% to 4.25% in permanent dentition, with a discrete predominance in women. The individuals with agenesis that most often seek treatment are those whose anterior teeth, especially lateral incisors, are missing^4^.

The absence of a lateral incisor is usually diagnosed early, during mixed dentition, or even in adolescence, when parents seek treatment for their children because of aesthetic concerns. 

There are three options for the replacement of MLI: replacement with a canine orthodontically moved and reshaped; dental implants; or tooth-supported restorations. The challenge here is to develop a comprehensive treatment plan according to diagnosis, age and the needs of each patient^4^.

#### 1. Replacement of missing MLI with a canine

The replacement of a MLI with a canine is a highly favorable option, because canines may be restored right after they have been moved orthodontically. Moreover, the presence of a natural tooth preserves and ensures the growth of the alveolar bone.

When planning, special attention should be paid to canine size, shape and shade. Canines are usually larger and buccolingually thicker, which may affect the tooth-size relationships in the anterior area. Therefore, some steps should be followed to ensure that it functions adequately as a lateral incisor:

1) The lingual surface of the canine should be reduced because of its buccolingual thickness.
2) The incisal edge should usually be flattened to reduce the cusp.
3) The proximal surfaces should be reduced, but special attention should be paid to avoid introducing interproximal ledges that may accumulate plaque.
4) The convex buccal surface should be flattened carefully to avoid darkening the tooth in case of excessive thinning of the enamel.
5) Individual bleaching should be performed whenever necessary.
6) Some extrusion may provide an adequate gingival margin.
7) Torque should be corrected to produce light reflection similar to that of the lateral incisor.
8) Mesioincisal and distoincisal angles should usually be restored using composite resin^3^ ^,^ ^4^ ^,^ ^10^ ^,^ ^11^.

Obviously, the smaller and lighter the canine is, the easier its reshaping into a lateral incisor will be. All the dimensions of large canines can be reduced, but sensitivity and darkening may increase because of enamel thinning. In some cases, a resin veneer should be used for a successful result. However, even the most challenging canines may be used to replace adjacent lateral incisors as long as the technique applied is correct and files are operated in high rotation under abundant water spray irrigation and mineralization procedures are applied at every visit^3^ ^,^ ^10^.

Along history, patients with agenesis of the lateral incisor have been classified as good candidates for canine replacement when they have Class II malocclusion with minimal mandibular crowding. Another favorable condition is the presence of crowding that requires extraction of mandibular premolars in patients with Class I malocclusion^3^ ^,^ ^9^.

Canine replacement with the premolar should also follow very strict criteria. First, the height of gingival margins should be examined to determine how much the first premolar should be intruded. As the premolar margins will become the gingival margins of the reshaped canine, they should be leveled with the gingival margins of central incisors. In some cases, the best results are achieved by increasing the crown by means of a gingivectomy. After that, the premolar may be restored using a composite resin or porcelain, which will change it into a canine with good esthetic and functional characteristics.

Moreover, a central incisor may also be replaced with a lateral incisor in case the central incisor is lost or missing. In this case, the lateral incisor is moved toward the midline and to the center of the space where the central incisor should be, then intruded to level gingival margins and later restored.

When implant placement is planned, it is necessary to wait for the completion of alveolar growth while preserving the space that will be necessary for later rehabilitation. In general, a graft is necessary before implant placement^3^ ^-^ ^6^. For some dentists, this treatment option is not good, because canine guidance cannot be achieved, which may lead to occlusal overloads on the premolars, as well as abfraction and loss of insertion. However, several studies have examined patients treated using canine replacement and have not found any significant differences in occlusal function or temporomandibular disorder^6^ ^-^ ^8^.

#### 2. Replacement of MLI with tooth implants

The replacement of a missing MLI with osseointegrated implants has some frequent complications. The alveolar process growth, more intense during patient growth but continuous along the whole life, does not stop as the patient ages. Therefore, this type of treatment often has unsatisfactory aesthetic results in the medium and long terms. Moreover, uprighting of incisors occurs as the patient gets older, and implants seem to become more protrusive.

However, implants in the aesthetic zone of patients with a high smile line are contraindicated because of the darkening of the gingival margin, reported in more than half of the patients after rehabilitation^12^. 

An argument in favor of using orthodontic movement to close the space is that possible complications of minimally invasive or noninvasive procedures are relatively easy to correct or repair, whereas treatments with implants are difficult or impossible to change afterwards. Despite the aesthetic complications observed in these cases, most patients with implants are apparently satisfied with the results of their treatment^4^ ^,^ ^13^.

## CASE 1

### CLASS II, DIVISION 2 MALOCCLUSION WITH CONGENITALLY MISSING MAXILLARY LATERAL INCISORS

The strategy used for the treatment and adjustment of smile aesthetics was orthodontic treatment with space closure, gingivoplasty and reshaping of maxillary canines to replace lateral incisors and of first premolars to replace the canines. The specialties involved in the treatment were Orthodontics, Periodontics and Restorative Dentistry.

**Figure 1:**
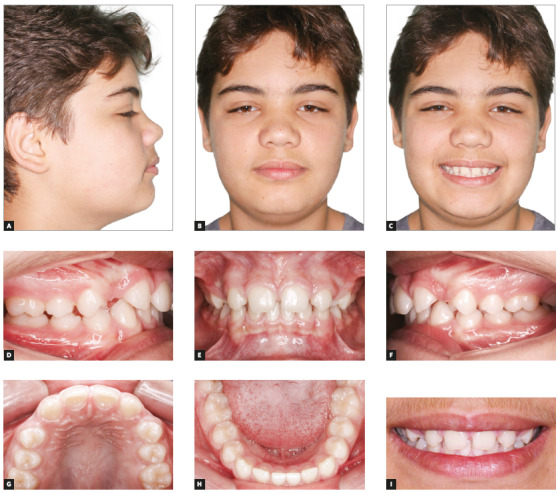
Initial facial photos (A-C); initial intraoral photos (D-H); close-up photo (I).

The patient was a 14-year-old adolescent with Angle’s Class II, division 2 malocclusion and congenitally missing teeth #12 and #22 (Fig 1). In the maxillary arch, canines erupted parallel and very close to the central incisors because of the congenital absence of lateral incisors. There were diastemas between incisors and canines, as well as wide spaces between canines and first premolars (Figs 1E, 1G). The periodontal phenotype was favorable, with excessive gingival displays and short crowns, both a result of altered passive eruption (Figs 1E, 1I). The mandible was well formed, with a slight curve of Spee and small diastemas between incisors. Overjet measured 6 mm and overbite was classified as deep (80%). The profile was slightly convex, the face was proportional, and there was good exposure of incisors when smiling (Figs 1A, 1B, 1C, 1I). Smile aesthetics was compromised by the anterior diastemas and the pointed canines in the place of lateral incisors. 

**Figure 2:**
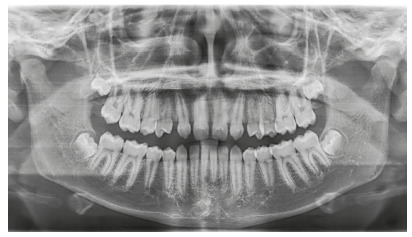
Initial panoramic radiograph.

After careful clinical analysis and examination of radiographs, photos and models, the choice was for orthodontic treatment and distalization of posterior maxillary teeth to close spaces in the maxillary arch. The choice to reshape canines to replace lateral incisors followed several parameters: Class II malocclusion, minimal mandibular crowding and facial balance; canine size, shape and color compatible with their reshaping into lateral incisors; short and narrow clinical crowns; no marked canine eminence in alveolar process. Canines are usually larger and buccolingually thicker, which may affect the dental relationships in the anterior segment, but this was not the case in this patient.

#### Multidisciplinary restorative aesthetic procedures (Periodontics, Restorative Dentistry)

At the end of the orthodontic treatment, the patient was referred to a periodontist for gingivoplasty to restore pink esthetics of the maxillary anterior area. After healing, the patient was referred to a Restorative Dentistry service. 

**Figure 3:**
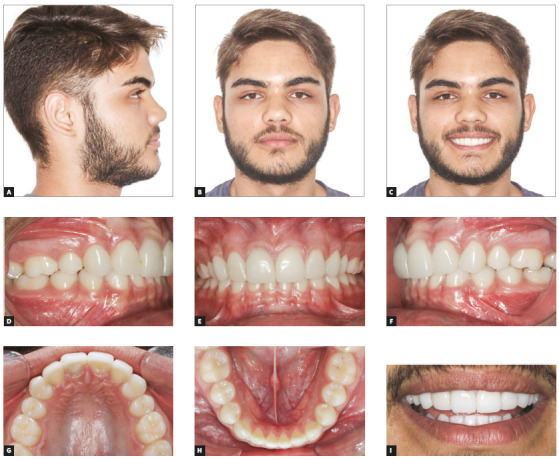
Final facial photos (A-C); final intraoral photos (D-H); close-up photo (I).

**Figure 4:**
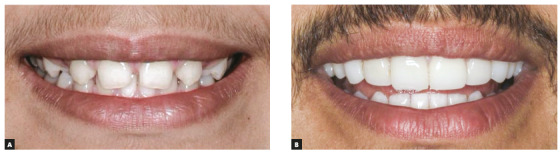
Close-up photos: A) initial; B) final.

Direct composite resin veneers changed the maxillary canines into lateral incisors. The same procedure was applied to central incisors. The fist premolars, in turn, were slightly reshaped, also using composite resin, and their shape eventually was very close to that of the canines. Such restorative procedures ensured occlusal balance and harmony, and smile aesthetics was satisfactory (Figs 3 and 4).

## CASE 2

### CLASS I MALOCCLUSION WITH CONGENITALLY MISSING TEETH #32 AND #42

The strategy used for the treatment and adjustment of smile aesthetics was orthodontic treatment to gain space for implants. The specialties involved in the treatment were Orthodontics, Implantology and Prosthetics. 

**Figure 5:**
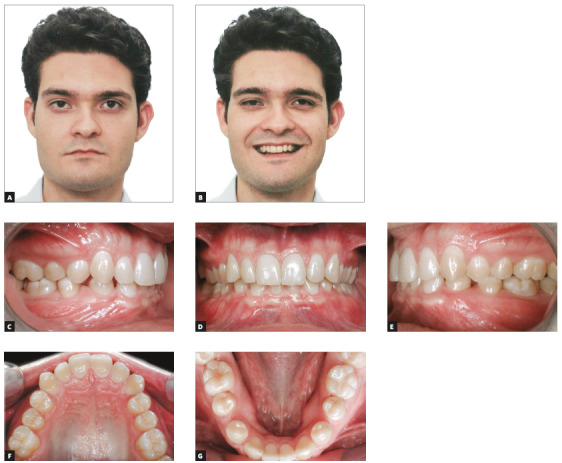
A, B) Initial facial photos; C-G) initial intraoral photos.

The patient was a 23-year-old young adult with Class I malocclusion and congenitally missing teeth #32 and #42 (Fig 5). Overjet measured 4 mm and overbite was classified as deep (80%). The examination of his face revealed a concave profile, brachycephalic face and good position of incisors when smiling (Fig 5). Smile aesthetics was slightly compromised by very wide buccal corridors. 

**Figure 6:**
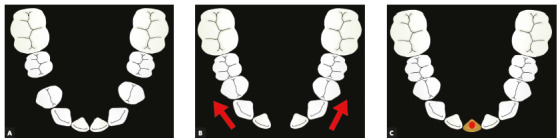
Drawing illustrating tooth movement in the mandibular arch. A) First stage; B) midline space opening; C) implant in place.

The mandibular arch had spaces between teeth #34 and #35, and #44 and 45, at a total of 6.5 mm. The maxillary arch was relatively well aligned, but with a specific feature: perceptible asymmetry between the size of teeth #11 and #21, as well as between #12 and #22. The Bolton analysis of the anterior area revealed a 7-mm excess in the maxillary arch. A maxillary excess was expected because there were six maxillary teeth and only four mandibular teeth. However, the amount of 7 mm was unexpected. Logically, the expected value should be close to or greater than 10 mm, because each lateral incisor measures a little more than 5 mm. The measurement in this case was explained by the reduced size of teeth #21 and #12, a factor that contributed to making up for this discrepancy. After detailed examination of clinical findings and radiographs, photographs and models, the treatment plan was orthodontic treatment with maximum anchorage, that is, total distalization of mandibular premolars and canines. This resulted in the opening of a space in the mandibular midline between teeth #31 and #41 (Figs 6A, 6B) for the placement of an osseointegrated implant to replace the incisor. The implant measured 6 mm, which was compatible with the size of the other incisors found in the mouth (Fig 6C). Bolton analysis indicated that the anterior mandibular segment needed only 7 mm of tooth structure to make it compatible with the anterior maxillary segment. To ensure that the implant to replace the incisor was not disproportionately large, interproximal reductions at a total of 1 mm were planned for the anterior segment of the maxillary arch. This involved the mesial and distal surfaces of teeth #11 and #22, originally larger than their contralateral teeth. This procedure would eliminate Bolton discrepancy and, at the same time, establish better symmetry for maxillary incisors. 

**Figure 7:**
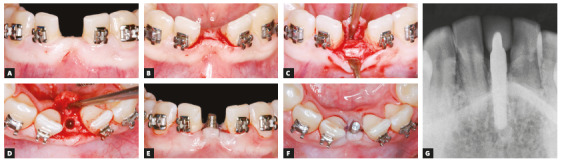
Sequence of placement between teeth #31 and #41 of one-piece osseointegrated implant. A) Anesthesia; B) incision; C) flap reflection; D) bone drilling; E, F) suture; G) periapical radiograph to evaluate implant position.

**Figure 8:**
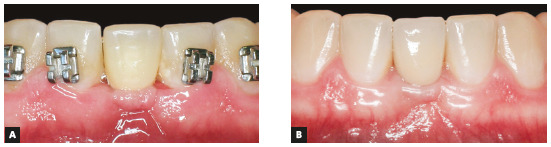
A) Provisional crown attached to implant; B) definitive crown attached to implant.

**Figure 9:**
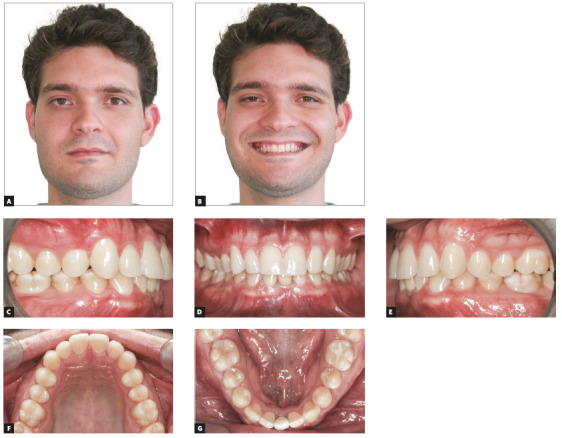
Final facial photos (A, B); final intraoral photos (C-G).

During the orthodontic treatment, the implant was placed after the correct leveling, alignment and opening of the space in the mandibular midline, between teeth #31 and #44. The implantologist chose a one-piece implant because of space limitations (Fig 7). The procedure was successful, and the implant was well positioned between the roots of teeth #31 and #41 (Fig 7G). This type of implant, as its name suggests, does not have any connections, and, therefore, it could be immediately loaded after the preparation of the coronal portion. After placement of the provisional (Fig 8A), it was kept free of masticatory loads for three months, until it was fully osseointegrated. After that, the implant was used in orthodontic procedures until treatment completion. At the end of the treatment, the provisional crown was replaced with a definitive crown (Fig 8B), and satisfactory aesthetics and function were achieved. Results revealed well-established occlusion and satisfactory levels of overbite and overjet, arch coordination and functional balance (Fig 9). 

## CASE 3

### CLASS III MALOCCLUSION WITH MISSING MAXILLARY CENTRAL INCISOR DUE TO TRAUMA

The strategy for treatment and correction of smile aesthetics was compensatory orthodontic treatment. Teeth #14, #34 and #44 were extracted, and the following teeth were reshaped: #22 to replace #21, #23 to replace #22, and #24 to replace #23. The specialties involved in the treatment were Orthodontics, Periodontics, Restorative Dentistry and Prosthetics.

**Figure 10:**
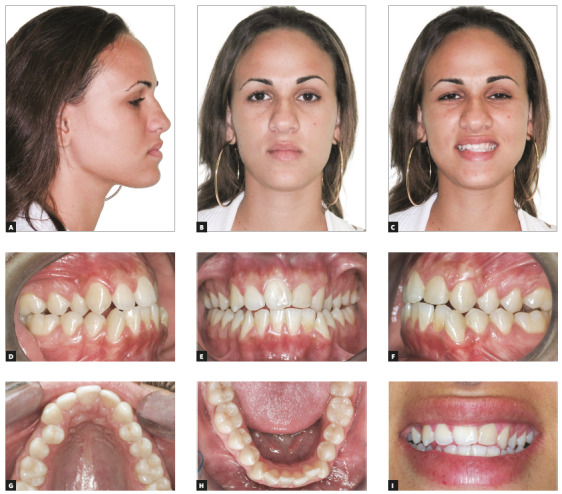
Initial facial photos (A-C); initial intraoral photos (D-H); close-up photo (I).

**Figure 11:**
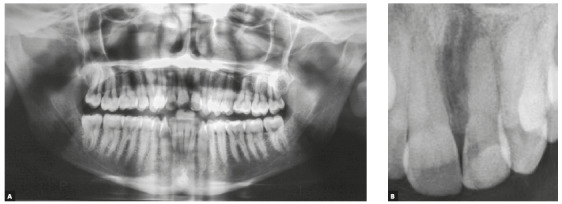
A) Initial panoramic radiograph. B) Initial periapical radiograph (tooth #22) shows complete pulp calcification.

The patient was a 24-year-old woman with severe maxillomandibular discrepancy (Class III) and Angle’s Class III malocclusion. Her left MCI was missing due to trauma at the age of 14 years, when there was also complete obliteration of the pulp chamber of tooth #22 (Fig 11B). Because of that, tooth #22 had a yellowish shade, which compromised her smile aesthetics substantially (Fig 10I). In addition to that, there was moderate crowding in the mandibular arch, and incisors were retroclined. Her left MLI was inclined mesially and in the place of the missing MCI, and there was a 3-mm midline shift to the left. Her profile was concave, her mandibular length was increased, and the menton was prominent (Figs 10A-10H). 

**Figure 12:**
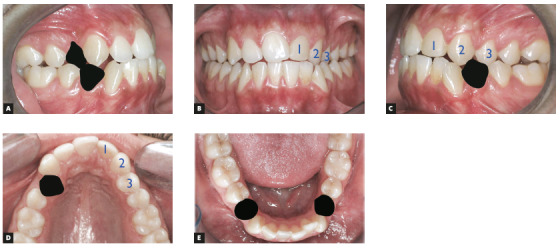
Treatment plan diagram. A) Extraction of teeth #14 and #44. B) Frontal view of teeth to be reshaped (#22, #23 and #24). C) Extraction of teeth #34 and lateral view of teeth to be reshaped (#22, #23 and #24). D, E) Maxillary and mandibular occlusal views of extractions and of teeth to be reshaped.

**Figure 13:**
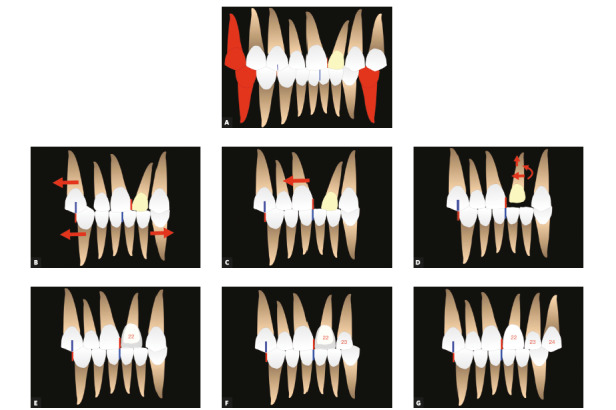
Treatment sequence. A) Extraction of teeth #14, #34 and #44; B) Distalization of tooth #13 completed, space for correction of maxillary midline; distalization of teeth #33 and #43, correction of anterior mandibular crowding and incisor retraction. C) Correction of maxillary midline; D) Mesial movement and intrusion of tooth #22. E) Provisional reshaping of tooth #22 to replace tooth #21. F) Provisional reshaping of tooth #23 to replace tooth #22. G) Provisional reshaping of tooth #24 to replace tooth #23.

A careful clinical analysis and examination of radiographs, photos and models indicated a compensatory orthodontic treatment, extraction of the two mandibular first premolars (#34 and #44) and the right maxillary first premolar, followed by reshaping of teeth #22, #23 and #24. Teeth were extracted in the mandibular arch to eliminate crowding and to promote the retraction of incisors to correct the anterior horizontal and vertical relationships of the arches using maximum anchorage. Two mini-implants were used for that purpose (Fig 15). In the maxillary arch, the extraction of the right first premolar and the placement of the mini-implant between the second premolar and the first molar reinforced the necessary anchorage for the retraction of tooth #13 and the correction of the midline An drawing illustrating the sequence of the treatment plan is shown in Figures 12 and 13.

**Figure 14:**
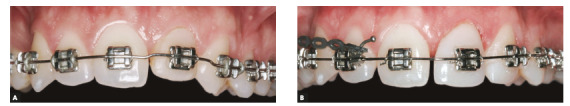
Provisional reshape of left maxillary lateral incisor to replace central incisor at beginning of treatment. A) Archwire bend for intrusion of tooth #22 to level gingival margin to that of tooth #11. B) Provisional reshape of tooth #22 to replace tooth #21.

**Figure 15:**
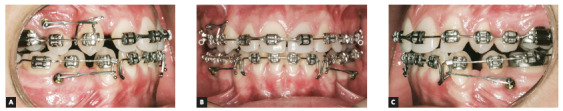
Treatment progression (20 months). A) TAD’s for retraction of teeth #13 and #43. B) Correction of maxillary midline. C) Retraction of tooth #33.

Early in the first months of treatment and after the intrusion of tooth #22 to level the gingival margin with that of tooth #11, the reshaping of the lateral incisor (#22) to replace the central incisor started, although still on a provisional basis (Fig 14). At the same time, reductions and polishing of the canine (#23) started to reshape it into a lateral incisor.

### Combined restorative aesthetic procedures (Periodontics, Restorative Dentistry)

**Figure 16:**
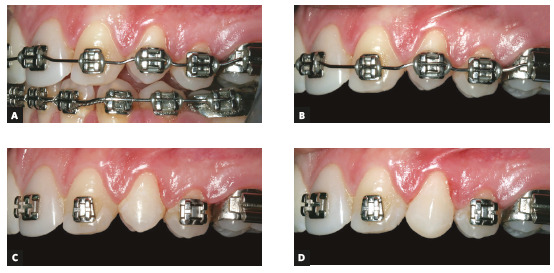
Reshaping of left maxillary first premolar to replace canine. A, B) Archwire bend for intrusion of tooth #24 for creation of interocclusal space for reshaping into canine and for leveling of gingival margin with that of left maxillary central incisor. C) Removal of brackets before restoration. D) Reshaping completed.

Close to the end of the treatment, the first premolar (#24) was intruded by adding bends to the archwire. The objective was to elevate the gingival contour and make it compatible with the canine height. Therefore, sufficient interocclusal space was created to reshape tooth #24 adequately and prepare it to replace the canine (#23). It should be kept in mind that these restorations were only provisional (Fig 16). 

After the completion of orthodontic movements, the second phase of restorations, the aesthetic phase, was initiated, and tooth bleaching was applied to both dental arches. 

**Figure 17:**
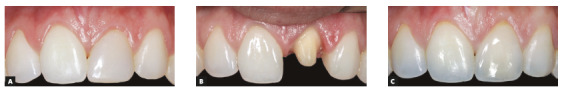
Tooth #22 restoration. A) Provisional restoration. B) Preparation of full crown in porcelain. C) Final porcelain crown after gingivectomy and adequate gingival recontouring.

One week after tooth bleaching was completed, treatment was focused on tooth #21, that is, tooth #22 now replacing tooth #21, because there were changes in pink and white aesthetics. The analysis of pink aesthetics revealed that the gingival level of tooth #11 was different from that of tooth #21 (Fig 17A). In white aesthetics, there were changes in the color of tooth #21 because of two factors: resin restoration with an inadequate shade selection; and, more importantly, the insufficient brightness (dark) of the tooth color because of the calcification of the pulp chamber (Fig 17A). Therefore, the choice was for a total crown reduction of tooth #21 to obtain enough space for a total ceramic crown in the adequate color (Fig 17B). First, a provisional resin crown was placed in tooth # 21, and the patient was referred to a Periodontics service for gingivoplasty of this tooth. After maturation of the gingival tissue, the tooth was prepared again to the subgingival margin, and, immediately after that, a new provisional crown was placed. Ten days after gingival recontouring while using the provisional, a definitive crown was placed. Therefore, smile aesthetics was satisfactorily rehabilitated, and occlusal function, previously compromised, was restored (Fig 17C).

**Figure 18:**
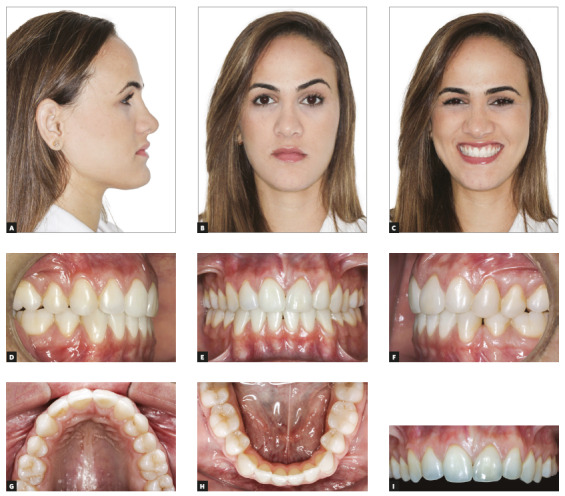
Final facial photos (A-C); final intraoral photos (D-H); close-up photo (I).

Treatment results indicated that the following objectives were achieved: correct leveling and alignment of arches, adequate intercuspation, correction of posterior crossbite and anterior open bite, establishment of adequate overbite and overjet. Maxillary midline was corrected, and the smile regained harmony and balance after reshaping of teeth #22, #23 and #24 according to strict proportion and shape principles. The retraction of mandibular incisors and the reduction of their inclination had a direct effect on the patient's profile, with a slight increase of facial concavity, which resulted in a harmonious relationship between lips, although there was an increase in the prominence of the menton. Tooth #22, which was reshaped to replace the central incisor (#21), resisted orthodontic movement well without any complications despite its complete pulp calcification already present before the beginning of the treatment (Figs 18A-I).

## BOLTON DISCREPANCY X EXTRACTION OF MANDIBULAR INCISORS

During orthodontic treatment, when extractions are necessary, the teeth most often selected are, in decreasing order: first premolars, second premolars and molars^14^. However, the indication of mandibular incisor extraction is sometimes the best treatment option. Significant Bolton discrepancy in the anterior area, that is, anterior mandibular excess, is the most important sign to justify exactly this procedure, and it is the main reason for this indication^15^ ^,^ ^16^. Supernumerary mandibular teeth and shape anomalies, such as macrodontia of mandibular incisors or microdontia of maxillary lateral incisors, are clear examples of this dental volume discrepancy. The extraction of mandibular incisors, when correctly indicated, is an excellent treatment option with several advantages, such as preservation of intercanine distance, considerably shorter treatment time, excellent functional and aesthetic results with little profile changes^17^ ^,^ ^18^. 

In contrast, if it is not correctly indicated, the extraction of a mandibular incisor may lead to disastrous results, such as increased overbite, reopening of the extraction site, unsatisfactory posterior occlusion, mandibular crowding relapse and interdental papillae aesthetic impairment, as well as the compromise of smile aesthetics if the muscle contraction pattern when smiling exposes mandibular teeth excessively.

## CASE 4

### CLASS I MALOCCLUSION WITH SEVERE ANTERIOR MANDIBULAR CROWDING AND BOLTON DISCREPANCY (4.8-MM ANTERIOR MANDIBULAR EXCESS)

The strategy for the treatment and adjustment of smile aesthetics was corrective orthodontic treatment with extraction of tooth #31, interproximal reduction, and reshaping using composite resins. The specialties involved in the treatment were Orthodontics and Restorative Dentistry.

A 24-year-old man presented with Angle’s Class I malocclusion, severe crowding in the maxillary and mandibular anterior areas, and tooth-size discrepancy with anterior mandibular excess of 4.8 mm. His facial profile was slightly convex, and his facial structures were balanced and harmonious. Incisors were adequately exposed when smiling, and his smile was relatively harmonious, except for the width of buccal corridors and exposure of crowded mandibular incisors.

**Figure 19:**
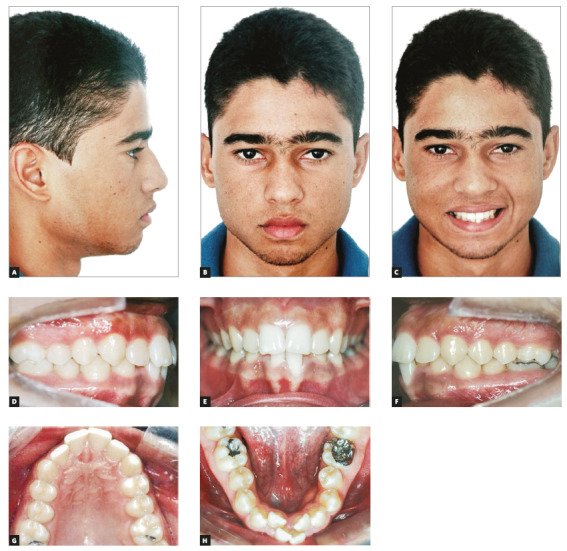
Initial facial photos (A-C). Initial intraoral photos (D-H).

Periodontal examination revealed that tooth #31 had extensive gingival recession on the buccal surface. The patient reported difficulty to clean his teeth because of the anterior mandibular crowding (Fig 19). 

During planning, extraction of tooth # 31 was suggested as the main alternative for the treatment of the 4.8-mm anterior mandibular Bolton discrepancy. This would result in adequate leveling, alignment and arch coordination to improve aesthetics and functioning. Tooth #31 was extracted because it was the smaller incisor (5.4 mm), and because it was malpositioned. An anterior mandibular excess of 0.6 mm would be created if 5.4 mm were removed from the anterior mandibular area, but that would be easily compensated for by interproximal reductions of teeth #11 and #21.

The treatment selected was based on the diagnostic setup, which confirmed the feasibility of that option and reinforced that the extraction of the mandibular incisor was an excellent treatment choice. 

Therefore, the treatment included the extraction of tooth #31 and, after that, the placement of a standard 0.022 x 0.028-in Edgewise fixed appliance. During leveling and alignment, both arches were expanded to decrease buccal corridors and, consequently, substantially improve smile aesthetics. 

Close to the end of the treatment, the mesial and distal surfaces of MCI required some small interproximal reductions to adjust the proportions of the maxillary and mandibular anterior segments. 

After orthodontic finishing and removal of the appliance, the patient was referred to a Restorative Dentistry service because of aesthetic details of the maxillary anterior area, such as the adjustment of incisal edges and embrasures, using reductions, composite resin additions, or both. 

**Figure 20:**
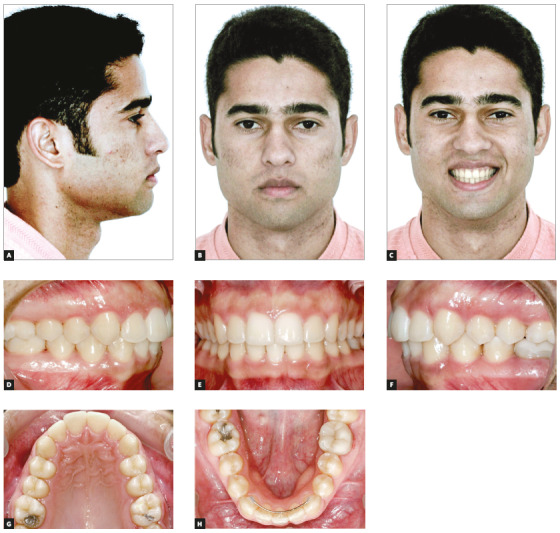
Final facial photos (A-C). Final intraoral photos (D-H).

The evaluation of results revealed well-established occlusion with adequate levels of overbite and overjet and satisfactory arch coordination. There were no perceptible changes in the patient’s profile, but the frontal view showed evident improvement of smile aesthetics. The most important result in cases that include the extraction of a mandibular incisor is adequate overbite and overjet (Figs 20D to 20F). For that reason, an accurate diagnosis is of utmost importance in these cases. The proportion between maxillary and mandibular arches is key for the success of these cases (Fig 20A-20H).

## FINAL CONSIDERATIONS

The results of increasingly frequent multidisciplinary orthodontic treatments of complex cases seem to effectively maximize aesthetic and functional results using a combination of procedures conducted by specialists in related areas, such as Surgery, Prosthetics, Implantology, Restorative Dentistry and Periodontics.

Therefore, coordinated actions to achieve aesthetic excellence in atypical cases requires fine tuning of a multidisciplinary team whose affinity, spirit of participation and technical quality should go hand in hand, and for whom patient satisfaction and excellence of results are the main objectives.
